# Increased Risk of Myositis-Specific and Myositis-Associated Autoantibodies After COVID-19 Pandemic and Vaccination: A Spanish Multicenter Collaborative Study

**DOI:** 10.3390/biomedicines12122800

**Published:** 2024-12-10

**Authors:** Laura García-Bravo, Alvaro Prada, María Gutiérrez Larrañaga, Eduardo Espinosa Ros, Delia Almeida González, Dolores Martín Martínez, Telesforo Rodríguez Sánchez, Carlos Gustavo Mingorance Gámez, Aurora Jurado Roger, Rocío Aguado Álvarez, María De Las Mercedes Díaz Luna, Carmen Rodríguez Hernández, Raquel de la Varga-Martínez, María López-Cueto, Maria Rosa Julià Benique, Miriam San José-Cascón, Bibiana Quirant-Sánchez, Alba Martínez-Chamorro, Goitzane Marcaida-Benito, Pilar Teresa Timoneda Timoneda, Marta Fandos Sánchez, Beatriz Sacristán Enciso, Kauzar Mohamed Mohamed, Teresa Guerra-Galán, Ángela Villegas, Andrés Roncancio-Clavijo, Margarita Rodríguez-Mahou, Silvia Sánchez-Ramón, Miguel Fernández-Arquero, Gloria Candelas-Rodríguez, Juliana Ochoa-Grullón, on behalf of the GEAI-SEI Group

**Affiliations:** 1 Immunology Department and IdISSC, Hospital Clínico San Carlos, 28040 Madrid, Spain; 2 Immunology Department, Hospital Universitario Donostia, 20003 San Sebastián, Spain; 3 Immunology Department, Hospital Universitario Nuestra Señora de Candelaria, 38010 Santa Cruz de Tenerife, Spain; 4 Immunology Department, Hospital Universitario Reina Sofía, 14004 Córdoba, Spainrocio.aguado.alvarez.sspa@juntadeandalucia.es (R.A.Á.); 5 Immunology Department, Hospital General Universitario Gregorio Marañón, 28007 Madrid, Spain; 6 Immunology Department, Hospital Universitario Puerta del Mar, 11009 Cádiz, Spain; 7 Immunology Department, Hospital Universitario Son Espases and Health Research Institute of the Balearic Islands (IdISBa), 07120 Palma de Mallorca, Spain; 8 Immunology Department, Hospital Universitario Germans Trias y Pujol, 08916 Badalona, Spain; 9 Immunology Department, Hospital Universitario de Jaén (HUJ), 46014 Valencia, Spain; 10 Immunology Department, Hospital General Universitario de Valencia, 46014 Valencia, Spain; 11 Immunology Department, Hospital de Mérida, 06800 Mérida, Spain; 12 Rheumatology Department, Hospital Clínico San Carlos, 28040 Madrid, Spain

**Keywords:** myositis autoantibodies, idiopathic inflammatory myopathies, anti-synthetase syndrome, anti-aminoacyl-tRNA synthetase autoantibodies, line blot immunoassays, coronavirus disease 2019, SARS-CoV-2 infection, COVID-19 vaccine, mRNA vaccine

## Abstract

**Background**: Emerging evidence suggests that SARS-CoV-2 infection and vaccines may trigger autoimmune responses in predisposed individuals. Idiopathic inflammatory myopathies (IIMs) are diseases with diverse clinical manifestations, often associated with myositis autoantibodies (MAs). Diagnosing IIM is challenging due to limitations in classification criteria and diagnostic assays. This study aimed to describe the incidence of IIM following SARS-CoV-2 infection or vaccination and compare rates between exposures. **Methods**: A multicenter observational study was conducted with 788 patients from 11 Spanish referral centers. A total of 1209 autoantibodies including myositis-specific autoantibodies (MSAs) and myositis-associated autoantibodies (MAAs), were analyzed using line blot immunoassay (LIA). **Results**: The study identified distinct patterns in aminoacyl-tRNA synthetase (ARS) antibody frequencies compared to pre-pandemic periods. Anti-PL-7 was the most prevalent ARS antibody (14.85%), while anti-Jo-1 was less frequent (7.23%). Anti-MDA5, commonly linked to SARS-CoV-2 infection, was detected in 11.68%. ANA positivity was observed in 60.66%, suggesting an autoimmune background. The most frequent diagnoses were anti-synthetase syndrome (ASSD) or IIM-non-ASSD (21.31%), followed by other systemic autoimmune diseases (SAIDs) (13.57%). Among the cohort, 91.13% received at least one dose of a messenger RNA (mRNA) COVID-19 vaccine, with a median of three doses per patient. Patients with prior SARS-CoV-2 infection or heterologous vaccination showed a higher frequency of multiple autoantibody positivity (*p* < 0.05), reflecting distinct immune signatures. **Conclusions**: This study provides valuable insights into the autoimmune risks and phenotypes associated with SARS-CoV-2 infection and vaccination, establishing a basis for further research on IIM and its link to MSAs and MAAs.

## 1. Introduction

Idiopathic inflammatory myopathies (IIMs) encompass a diverse group of autoimmune disorders with variable clinical manifestations. IIMs are primarily known for affecting skeletal muscles, causing chronic inflammation and progressive muscle weakness. Nevertheless, it is now recognized that IIMs can present without myositis, manifesting instead in the skin, lungs, heart, and joints or overlapping with other systemic autoimmune diseases (SAIDs) [1,2]. While the precise cause of IIMs is not yet fully understood, a combination of genetic and environmental factors, including viral infections, has been suggested as a potential trigger for their development [3].

Myositis autoantibodies (MAs), including myositis-specific autoantibodies (MSAs) and myositis-associated autoantibodies (MAAs), are considered key biomarkers of IIMs, with a prevalence of approximately 70% in patients, helping to classify patients into more homogeneous clinical phenotypes [4]. Anti-synthetase syndrome (ASSD), a subset of IIMs, is characterized by the presence of anti-aminoacyl-tRNA synthetase (ARS) autoantibodies and the clinical triad of arthritis, myositis, and interstitial lung disease (ILD). The pathophysiology of this condition involves an autoimmune response against cellular components, resulting in inflammation and tissue damage [5].

The diagnosis of IIMs is challenging due to the diversity of clinical manifestations and the wide range of MAs involved, as well as the lack of consensus on classification criteria and interpretation of autoantibody positivity [6]. For instance, the identification of MAs in patients with severe ILD has substantial implications for diagnosis and treatment, particularly in amyopathic dermatomyositis (DM) with anti-MDA5 autoantibodies and in cases of ASSD-associated anti-PL-7 or anti-PL-12 ARS autoantibodies [7,8].

Coronavirus disease 2019 (COVID-19) is a well-known multisystemic disease with a wide range of clinical manifestations, the first cases of which were reported in late 2019 in the Caucasian population. Emerging evidence suggests that the acute inflammatory response and production of autoantibodies contribute to the morbidity observed in patients with COVID-19 [9]. Recently, case reports in the literature have described more atypical and rarer presentations of infection with Severe Acute Respiratory Syndrome Coronavirus 2 (SARS-CoV-2) resulting in patterns of ILD [10]. Some pathologies can radiologically mimic this pulmonary involvement, as occurs in many SAIDs, such as ASSD, DM, polymyositis (PM), and systemic sclerosis (SSc) [11]. Therefore, performing an accurate differential diagnosis, including the determination of MAs, is crucial.

In recent years, the COVID-19 pandemic has also led to increased research interest in autoimmunity. Although rare, cases of IIM following SARS-CoV-2 infection or vaccination have been reported. Several studies to date suggest that more severe autoimmune reactions may manifest through mechanisms such as molecular mimicry or virus- or vaccine-induced Toll-like receptor activation [12]. A small but significant number of studies have reported the appearance of MAs after SARS-CoV-2 infection or vaccination. MAs detected include anti-Jo-1, anti-MDA5, anti-Mi-2α/β, anti-NXP2, anti-SAE1, anti-PL7, and anti-PL12 [13]. There are documented cases in the literature of DM, PM, and clinically amyopathic DM developing within months of SARS-CoV-2 infection [14]. Similarly, a small number of patients have presented with IIM symptoms shortly after receiving a COVID-19 messenger RNA (mRNA) vaccine [15].

Preliminary results showed an increased incidence of anti-MDA5 antibodies coinciding with the onset of SARS-CoV-2 infection in 2020, as well as anti-PL7 antibodies at the time of COVID-19 vaccination in 2021 [16]. Given the need for further epidemiological investigation, a multicenter study was conducted to assess the possible association of MAs, especially MSA-ARS autoantibodies, with SARS-CoV-2 infection and/or vaccination. For this purpose, patients with a specific autoantibody profile were studied.

## 2. Methods

### 2.1. Patients and Study Design

A multicenter retrospective observational study was conducted by the Immunology Department of the Hospital Clínico San Carlos in Madrid, Spain. Serum samples from patients with suspected IIM were referred to the immunology laboratories for MAs determination by line blot immunoassay (LIA). Further analysis was performed on samples that tested positive for one or more Mas, and these samples were collected between January and December 2022.

The results of the other parameters examined were obtained from the same or a closely related day’s autoantibody analysis. A patient was considered to have a SARS-CoV-2 infection if they tested positive using reverse transcription polymerase chain reaction (RT-PCR) or a SARS-CoV-2 antigen test, with written evidence of this in the patient’s medical record. The vaccines administered were classified into two groups. mRNA vaccines included the mRNA-1273 SARS-CoV-2 vaccine (Moderna, Cambridge, MA, USA) and the BNT162b2 vaccine (Pfizer, New York, NY, USA). Non-mRNA vaccines included the ChAdOx1 nCoV-19 vaccine (Oxford/AstraZeneca, Cambridge, UK) and the Ad26.CoV2.S vaccine (Janssen, Beerse, Belgium). The term “combined vaccine” refers to administering both types of vaccines to the same patient.

For a more rigorous statistical analysis of the clinical associations and implications for diagnosis and prognosis, MAs were divided into three groups: 1. *MSA-ARS* (myositis-specific anti-aminoacyl-tRNA synthetase autoantibodies), including anti-Jo-1, anti-PL-7, anti-PL-12, anti-EJ, and anti-OJ); 2. *MSA- Non-ARS* (myositis-specific non-anti-aminoacyl-tRNA synthetase autoantibodies), including anti-SRP, anti-Mi-2α/2β, anti-MDA5, anti-NXP2, anti-TIF1-γ, and anti-SAE autoantibodies; and 3. *MAA* (myositis- associated autoantibodies) including anti-Ro-52, anti-Ku, and anti-PM-Scl-75/100. Similarly, patients were classified into three groups based on their autoantibody profile: (1) *Group I: MSA-ARS patients* (includes patients with one MSA-ARS and those with one MSA-ARS combined with one MAA); (2) *Group II: MSA-Non-ARS patients* (includes patients with one MSA-Non-ARS, patients with one MAA and patients with one MSA-Non-ARS combined with one MAA); and (3) *Group III: Multi-specificity patients* (includes patients with two or more positive MAs, excluding those belonging to groups I and II).

In accordance with the clinical manifestations, patients were classified based on their clinical examination and evaluation by their physicians. These classifications included asthenia, muscular involvement (proximal symmetrical weakness, motility disorders), skin lesions (heliotrope rash, Gottron’s papules/signs, mechanic hands), and pulmonary involvement (clinical findings, high-resolution computed tomography patterns, pulmonary function test results, or lung biopsy findings), and/or the presence of neoplasia. It is important to note that patients with concomitant infections unrelated to SARS-CoV-2 were excluded from the study to minimize potential confounding factors. Additionally, oncological variables and baseline autoimmune disease characteristics were systematically clustered for analysis.

Finally, patients were divided according to their final diagnosis into four main groups: 1. *ASSD or IIM-non-ASSD* (including PM, DM, and immune-mediated necrotizing myopathy); 2. *SAID* (including systemic lupus erythematosus (SLE), Sjögren syndrome (SjS), SSc, or rheumatoid arthritis (RA), among others); and 3. *Overlap IIM-SAID*. The criteria used were those proposed by Solomon for ASSD and those proposed by EULAR/ACR for patients with IIM-non-ASSD and SAID. Patients whose clinical features were specific but insufficient for an accurate diagnosis were classified as pre-ASSD, pre-IIM-non-ASSD, pre-SAID, or pre-IIM/SAID.

The study was approved by the Research Ethics Committee (REC) of Hospital Clínico San Carlos (approval code: 22/534-E) and was conducted in compliance with the requirements established under the current legislation, specifically Royal Decree 1090/2015. Given the retrospective and multicenter design of the study, the ethics committee granted an exemption from obtaining informed consent.

### 2.2. Laboratory Assessment

#### 2.2.1. Line Blot Immunoassay Analysis

In patients with clinical suspicion of IIM, MAs were analyzed using LIA methods from three commercial companies. The EUROIMMUN Myositis blot (AG, Lübeck, Germany) was used by 9 of the 11 centers and the following 16 myositis autoantibodies were measured: Jo-1, PL-7, anti-PL-12, EJ, OJ, SRP, Mi- 2α, Mi-2β, MDA5, NXP2, TIF1-γ, SAE 1, Ro-52, Ku, PM-Scl-75, and PM-Scl-100. The intensities of the strips were analyzed using the EUROLineScan system (version 3.2.6.20), and semi-quantitative units were obtained. According to the manufacturer’s recommendations, the detection limit for weak positive results was set at 11.

The other two centers employed a myositis blot from two distinct commercial entities. The BlueDot Myositis MYO12D-24 (D-TEK, Beerse, Belgium) measures the following twelve MAs: Jo-1, PL-7, PL-12, EJ, SRP, Mi-2, MDA-5, TF1γ, HMGCR, Ro52, SAE1/SAE2, and NXP2. In addition, the following eight MAs were measured by Palex (Sant Cugat del Vallès, Barcelona): Jo-1, PL-7, PL-12, SRP, Mi-2, Ku, PM-Scl, and Scl-70. The assays yielded positive results with lower cut-off points of 10 for both assays. As can be observed, the autoantibodies were measured in a similar manner, with the exception of the autoantibody pairs PM75/PM100 and Mi-2α/Mi-2β, which were measured together. Due to the observed discrepancies in antibody measurement, it was deemed necessary to homogenize the data by quantifying the Mi-2α/Mi-2β and PM/Scl-75/PM-Scl-100 antibody pairs collectively.

A standard level of band intensity positivity was established for all centers, with low positivity (LPOS) < 20 and high positivity (HPOS) > 20 being considered.

#### 2.2.2. Indirect Immunofluorescence Assay

Antinuclear antibody (ANA) detection was performed by the indirect immunofluorescence (IIF) technique using human epithelial cell line (HEp-2) substrate slides. The serum samples were titrated from a starting dilution of 1/80 to the endpoint, with the results expressed as the last positive dilution. The ANA patterns observed were defined according to the International Consensus on ANA Patterns (ICAP). The results were stratified according to the titer positivity grouped into three categories: low titer (1/80 and 1/160), medium titer (1/320), and high titer (1/640 or ≥1/1280).

#### 2.2.3. Extractable Nuclear Antigen Antibodies

Following a positive ANA test, extractable nuclear antigen (ENA) antibody screening was evaluated using two different immunoassays among the participating centers: the BioPlex 2200 ANA screen (Bio-Rad Laboratories, Hercules, CA, USA) and the APTIVA (CTD Essential, Werfen, Bedford, MA, USA). The detected anti-ENA antibodies not included in the myositis blot were anti-DNA Topoisomerase I (anti-Scl-70), anti-double-strand DNA (anti-dsDNA), anti-La/SSB, anti-Ro60/SSA, anti-Sm, and anti- ribonucleoprotein (anti-RNP).

#### 2.2.4. Muscle and Liver Enzymes

Measurement of serum levels of muscle creatine kinase (CK), alanine aminotransferase (ALT), aspartate aminotransferase (AST), and lactate dehydrogenase (LDH) was performed using an immunoturbidimetric method (Beckman Coulter AU480 Analyzer, Brea, CA, USA). The reference range for CK is ≤155 U/L, for ALT it is ≤35 U/L, for AST it is ≤35 U/L, and for LDH it is 208-378 U/L. On the other hand, aldolase was quantified using a colorimetric enzymatic method (Sentinel Diagnostic, Milan, Italy), with normal reference values of 1.0–7.6 IU/L. Levels above this range were classified as abnormal.

#### 2.2.5. Analysis of HLA Typing

The LIFECODES HLA-SSO kit (Immucor, Inc., Norcross, GA, USA) was used for low-resolution typing of human leucocyte antigen (HLA) class I (HLA-A and HLA-B) and class II (HLA-DRB1, HLA-DQA1 and HLA-DQB1) alleles. Microsphere-bound oligonucleotide probes were analyzed using the Luminex 100/200 system (Luminex Corp., Austin, TX, USA), a flow cytometry-based platform using the principles of xMAP technology. Written informed consent for genetic analysis was obtained from the patients studied.

### 2.3. Statistical Analysis

The statistical analysis was conducted using SPSS software version 27, Microsoft Excel version 14.1.0, and GraphPad Prism version 8.1.0. Continuous variables are reported as medians and interquartile ranges (IQRs) due to the non-normality of their distribution, while categorical variables are described as frequencies and percentages. The chi-square test was employed to compare categorical variables, with statistical significance set at *p* < 0.05.

## 3. Results

### 3.1. Overall Overview and Incidence of Myositis Autoantibodies

A retrospective analysis was performed on patients’ samples in which an MA profile was requested between January 2022 and December 2022. If a patient underwent one or more repeated tests with a positive MA result after an initial negative result, only the initial positive result was considered.

A total of 11 hospitals in Spain participated, with the following distribution of patients in descending order: Hospital Universitario Donostia, San Sebastián (n = 250); Hospital Clínico San Carlos, Madrid (n = 230); Hospital Universitario Nuestra Señora de Candelaria, Santa Cruz de Tenerife (n = 154); Hospital Universitario Reina Sofía, Córdoba (n = 50); Hospital General Universitario Gregorio Marañón, Madrid (n = 31); Hospital Universitario Puerta del Mar, Cádiz (n = 19); Hospital Universitario Son Espases, Palma de Mallorca (n = 17); Hospital Universitario Germans Trias i Pujol, Badalona (n = 15); Hospital Universitario de Jaén (HUJ) (n = 14); Hospital General Universitario de Valencia (n = 4); and Hospital de Mérida (n = 4).

A total of 841 patient requests were received out of more than 1000 positive patients reported. After applying the exclusion criteria, the total number of patients included was 788 with a cumulative count of 1209 MAs. Repeated requests (n = 50) for the same patient were excluded. Patients under 18 years of age were also excluded.

**Figure 1:**
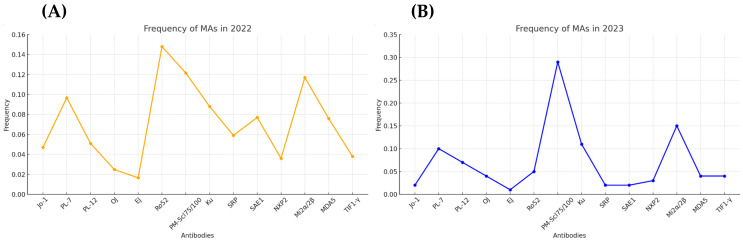
Frequency of myositis autoantibodies (MAs) in years 2022 (**A**) and 2023 (**B**).

The frequency distribution of each MA collected in this study, corresponding to the 788 patients included, is shown in Figure 1A. To compare whether the positivity profile was similar in subsequent years, the frequency of MAs in 2023 was calculated using patients from the San Carlos Clinical Hospital as a cohort (Figure 1B).

Table 1 summarizes the demographic, clinical, and laboratory characteristics of each patient group, which will be discussed throughout the manuscript. Regarding demographic data, the median age of the patients was 61 years (IQR: 50–71), with a female predominance (n = 523, 66.37%) over males (n = 265, 33.63%). The majority of patients referred for MA testing were from rheumatology or pneumology departments (n = 552, 70.05%), while the remaining (n = 236, 29.94%) were referred from other specialists. At the time of the study, 18 patients (2.28%) had died, 66.67% were associated with oncological disease, 27.78% with SAID, and 16.67% with IIM, two of whom had anti-TIF1-γ antibodies.

### 3.2. Autoantibody Profile: Frequency and Levels

**Figure 2:**
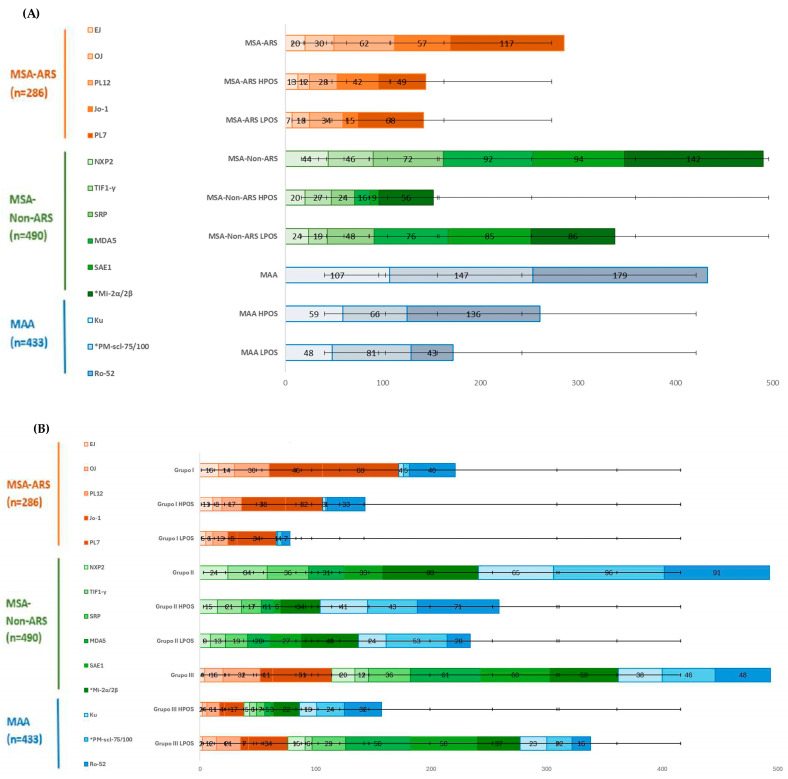
Frequency and levels of myositis autoantibodies (MAs) adapted from an image of Euroimmun [17]. (**A**) Distribution of MAs grouped by antibody type. (**B**) Distribution of patient groups classified by MA profile. Results of MAs measured by line blot immunoassay are represented in ascending order of global frequency. Myositis-specific anti-aminoacyl-tRNA synthetase autoantibodies (MSA-ARS) including anti-EJ, anti-OJ, anti-PL12, anti-Jo-1, and anti-PL7 are shown in orange. Myositis-specific non-anti-aminoacyl-tRNA synthetase autoantibodies (MSA-Non-ARS), including anti-NXP2, anti-TIF1-γ, anti-SRP, anti-MDA5, anti-SAE1, and anti-Mi2α/2β, are shown in green. Myositis-associated autoantibodies (MAA), including anti-ku and anti-PM-scl-75/100, are shown in blue. HPOS and LPOS were used to refer to high and low positivity, respectively, in the myositis panel.

Figure 2A illustrates the total number of MAs, along with their respective positivity levels expressed in absolute numbers. Of the total 1209 MAs, 286 (23.65%) were MSA-ARS, 490 (40.52%) were MSA-Non-ARS, and 433 (35.81%) were MAA. The most prevalent MA was anti-Ro-52 (14.81%), followed by anti-PL-7 (9.68%), with the *anti-PM-Scl-75/100 and *anti-Mi-2α/β antibody pairs accounting for 12.16% and 11.75%, respectively. A total of 557 (46.07%) HPOS autoantibodies were observed, distributed in 144 MSA-ARS (25.85%), 152 MSA-Non-ARS (27.29%), and 261 MAA (46.89%). The HPOS MAs, in descending order of frequency, were as follows: anti-Ro-52 (24.42%), anti-Ku (10.59%), anti- Mi-2α/β (10.05%), and anti-PL7 (8.77%). On the other hand, the total number of LPOS was 652 (53.93%), grouped into 142 MSA-ARS (21.78%), 338 MSA-Non-ARS (51.84%), and 172 MAA (26.38%). The most common LPOS MA, listed in ascending order of frequency, was anti-SAE1 (13.04%), *anti-Mi-2α/β (13.19%), *anti-PM-Scl-75/100 (12.42%), anti-MDA5 (11.66%), and anti-PL-7 (10.43%).

As illustrated in Figure 2B, the 788 patients were categorized into three groups based on their autoantibody profile. Group I included 123 MSA-ARS (15.61%) and 49 MSA-ARS + MAA (6.22%). Group II comprised 193 MSA-Non-ARS (24.50%), 201 MAA (25.51%), and 49 MSA-Non-ARS + MAA (6.22%). Group III consisted of 173 patients (21.95%) with various combinations of autoantibodies, including 114 MSA-ARS, 248 MSA-Non-ARS, and 134 MAA. In Group I, the most prevalent MSA-ARS was anti-PL7 (38.37%), followed by Jo-1 (26.74%). The most prevalent associated MAA in this subgroup was Ro-52 (81.63%), positive in 23.26% of MSA-ARS patients. In Group II, the most prevalent MSA-Non-ARS was anti-Mi-2α/β (16.87%), with the associated MAAs being PM-Scl-75/100 (38.09%) and Ro-52 (36.11%). The remaining MSA-Non-ARS autoantibodies exhibited a similar frequency.

As shown in Table 1, there were significant differences in the antibody titers between the groups. In particular, the levels of HPOS autoantibodies were found to be significantly higher in Groups I and II compared to Group III (*p* < 0.05). Conversely, patients in Group I exhibited greater HPOS autoantibodies than those in Group II (*p* < 0.05). Among patients in Group I with HPOS, the most prevalent MAs were Jo-1 (26.857%), Ro52 (23.07%), and PL-7 (22.37%). In groups II and III, the most prevalent autoantibody was anti-Ro-52, present in 27.41% and 20.38% of cases, respectively. Regarding LPOS autoantibodies, PL-7 was the most prevalent autoantibody in Group I (43.59% of cases), PM-Scl-75/100 in Group II (22.65%), and SAE1 and MDA5 in Group III, accounting for 17.16% and 16.57% of cases, respectively.

### 3.3. MAs Associated with ANA Antibodies

In regard to ANA-associated autoantibodies, 60.66% (n = 478) of patients exhibited a positive result, with no statistically significant differences between groups. In Group I, 63.37% of ANA-positive cases displayed a cytoplasmic pattern, which was significantly higher than those observed in Groups II and III (*p* < 0.05). Among the MSA-ARS autoantibodies in Group I, the predominant IIF pattern for Jo-1 was the cytoplasmic fine speckled (AC-20) pattern (54.76% of positive cases), whereas the cytoplasmic fine dense speckled (AC-19) pattern was mostly observed in association with PL-12 (53.85% of positive cases). The nuclear fine speckled (AC-4) pattern was identified as the predominant pattern for the remaining MSA-ARS autoantibodies (PL-7, EJ and OJ). In contrast, in Groups II and III, the predominant ANA pattern was nuclear fine speckled (AC-4), observed in 84.49% and 73.87% of cases, respectively, which was significantly higher than in Group I (44.95%, *p* < 0.05).

Finally, regarding the presence of ENAs, 17.13% of patients tested positive, with no significant differences observed between groups (*p* > 0.05). Among these, the most prevalent antibody was the Ro52 (65.98%) associated with ASSD (27.83%). Referring to the ENAs not included in the myositis blot, RNP was the most prevalent antibody (12.92%), predominantly associated with ILD in 31.57% of cases, particularly in the context of CTD, SLE, and SSj.

### 3.4. Antibody Association with COVID-19 Infection or Vaccination

The different combinations between groups according to whether they were infected and/or vaccinated against SARS-CoV-2 are shown in Table 1. Of the 788 total patients, 720 were vaccinated and distributed among the following groups: Group I (n = 165), Group II (n = 393), and Group III (n = 162) (*p* < 0.05 Group I vs. Group II). In the same way, there were a total of 299 patients with documented infection, distributed by group as follows: Group I (n = 60), Group II (n = 158), and Group III (n = 81) (*p* < 0.05 Group I vs. III).

Among patients with a history of SARS-CoV-2 infection, 47.16% of positive cases had onset of symptoms suggestive of IIM after infection. There was a greater number of patients who belonged to Group III compared to Group I (26.59% vs. 13.95%, *p* < 0.05). A total of 44 patients were diagnosed with long-COVID19, with 28 belonging to Groups I and II and 16 to Group III.

Referring to vaccinated patients, 43.33% had symptoms compatible with IIM after vaccination. In Group II there was a lower percentage of patients (36.57%) compared to Groups I and II (43.93% and 43.02%, respectively), but with no significant difference between groups (*p* > 0.05).

The distribution according to the type of vaccine administered indicated a higher proportion of mRNA vaccines in Groups I and III compared to Group II (Group II vs. III, *p* < 0.05). The group of patients with the highest non-mRNA vaccine administration was Group II (*p* > 0.05), while in Group III, the presence of combined vaccines was significantly greater (*p* < 0.05).

### 3.5. Description of Clinical Manifestations. Diagnosis, Treatment, and Outcomes

The clinical characteristics of each group of patients are presented in Table 1. The prevalence of asthenia was significantly higher in Groups I and III than in Group II (*p* < 0.05). Pulmonary involvement and the presence of a neoplasm were more prevalent in Group I than in Groups II and III (*p* < 0.05). A significant majority of patients with fibromyalgia were observed in Group II (*p* < 0.05). No significant differences were observed between the groups for the remaining clinical manifestations.

Patients were subsequently classified into categories based on their final diagnosis. A total of 168 patients (21.31%) were diagnosed with ASSD or IIM-non-ASSD, with significantly higher proportions in Groups I and III compared to Group II (*p* < 0.05). Additionally, 54 patients (6.85%) were classified as pre-ASSD/pre- IIM-non-ASSD, with this diagnosis being more frequent in Group I than in Group II (*p* < 0.05). Another group consisted of 107 patients (13.57%) diagnosed with systemic autoimmune diseases (SAIDs) other than IIM. The most common SAIDs were SLE (n = 15), RA (n = 14), and SjS (n = 11). Other diagnoses included vasculitis, connective tissue disease, antiphospholipid syndrome, celiac disease, multiple sclerosis, cryoglobulinemia, primary biliary cholangitis, and bullous pemphigus. Group III showed a significantly higher prevalence of these conditions compared to Group I (*p* < 0.05). Another group comprised 38 patients (4.82%) diagnosed with overlap IIM-SAID. Finally, the remaining 421 patients (53.42%) were classified as having no diagnosis or non-specific clinical features of IIM.

Table 2 presents the main characteristics of each patient group, categorized according to their final diagnosis. The first column lists the three most frequent antibodies in each group. Notably, the ASSD/IIM-non-ASSD group exhibited the highest count of HPOS antibodies (n = 63, 58.33%), with significant differences compared to the other groups (*p* < 0.05). The most prevalent MSA-ARS antibody in this group was anti-PL7 (n = 29, 17.26%).

However, in the pre-ASSD/IIM-non-ASSD group, the most frequent MSA-ARS antibody was anti-Jo-1 (n = 10, 18.51%). In both the SAID group and the overlap IIM-SAID group, the most frequent antibody was anti-Ro52 (37.38% and 34.21%, respectively). Among patients with non-specific IIM symptoms, the most prevalent antibodies were the anti-Mi2α/β and anti-PM-Scl-75/100 pairs (n = 81, 19.23% for both).

A total of 102 patients presented with an associated oncological condition. Notably, the antibodies most frequently associated with neoplasia were anti-Ro-52 (n = 28), anti-PM-Scl-75/100 (n = 19), and anti-TIF1-γ (n = 15). Among patients diagnosed with DM, 27.90% had elevated anti-TIF1-γ antibody titers, 66.67% of which were paraneoplastic. On the other hand, among the total number of patients with ILD (n = 123, 15.61%), the antibody most closely associated was anti-MDA5 (22.76%), followed by anti-Ro-52 (21.95%) and anti-SAE (21.95%). Among patients diagnosed with ASSD/IIM-non-ASSD with associated ILD, 30.45% had anti-MDA5 antibodies. Regarding skin involvement (n = 150, 19.06%), the most frequently associated antibodies were anti-Ro-52 (43.33%), anti-PM-Scl-75/100 (29.33%), anti-Mi-2α/β (27.33%), and anti-Ku (24%).

The distribution of ANA antibodies in the different groups is shown in Table 2. The presence of ANA was statistically higher (*p* < 0.05) in the ASSD/ IIM-non-ASSD (69.64%), SAID (75.70%), and overlap IIM-SAID (73.68%) groups compared to the pre-ASSD/ IIM-non-ASSD (59.25%) and mixed/non-specific (52.25%) groups.

Regarding the onset of symptoms following infection, a significantly higher proportion of patients were classified into the pre-ASSD/IIM-non-ASSD and SAID groups compared to other groups (*p* < 0.05).

In contrast, patients who developed symptoms suggestive of IIM after vaccination were distributed across all groups without significant differences (*p* > 0.05), although the highest percentage was observed in the SAID group (45.79%) compared to all vaccinated patients, with or without documented infection. Similar results were observed among patients who were only vaccinated without documented infection, as shown in Table 2, with the highest percentage found in the overlap IIM-SAID group (47.36%).

The comparison between patients with documented infection and those only vaccinated without documented infection revealed statistically significant differences in the prevalence of anti-TIF1-γ antibodies (*p* < 0.05) in the vaccinated group and a higher prevalence of anti-PM-Scl antibodies (*p* < 0.05) in the infected group.

### 3.6. HLA Typing in a Subgroup of Patients

A subset of 36 patients from the Hospital Clinic San Carlos underwent HLA class I and II gene analysis. All patients had a positive result for MA in 2022 and the result was confirmed one year later, along with the HLA gene study. While HLA testing was only available for a subset of the cohort, we deemed it was important to include the results in the article for the benefit of the wider readership.

Of the patients studied, 61.11% (n = 22) had a diagnosis of IIM/ IIM-non-ASSD or SAID, while the remainder (38.88%, n = 14) had at least one of the following clinical symptoms: ILD, arthralgia, neurological symptoms, or fibromyalgia. The main antibodies found were MSA-ARS, present in 50.00% (n = 18) of patients, the most common being anti-PL7 and anti-PL12 (n = 13 combined). With regard to HLA class I gene typing, all patients carried at least one of the following HLA-DRB1 alleles: HLA-DRB1*01, HLA-DRB1*03, HLA-DRB1*04, HLA-DRB1*07; HLA-DRB1*11; HLA-DRB1*13; and/or HLA-DRB1*15. The most common alleles were HLA-DRB1*03 and HLA-DR*04, present in 55.56% (n = 20) of all patients. The HLA- DRB1* allele combinations in the population were distributed as follows: 16.67% (n = 6) of patients were homozygous for one of the above alleles, 63.89% (n = 23) of patients carried combinations of two of these HLA-DRB1 alleles, and 19.44% (n = 7) carried only one of these alleles in combination with another allele.

In contrast, 88.89% (n = 32) of the patients carried at least one of the following HLA-A alleles in ascending order: the most common alleles were HLA-A*02 (44.44%, n = 16), HLA-A*24 (16.67%, n = 6), HLA-A*26 (13.89%, n = 5), HLA-A*30 (11.11%, n = 4), and/or HLA-A*33 (n = 5). It is worth noting that only two patients were homozygous. One was homozygous for HLA-A*26 and had been diagnosed with ASSD following SARS-CoV-2 infection. The other was homozygous for HLA-A*02 and had experienced severe neurological symptoms following SARS-CoV-2 infection. The results showed that 37.55% (n = 12) of patients carried two of the aforementioned alleles, while the remainder (62.55%, n = 20) carried only one.

Finally, the allele distribution for the HLA-B genes was found to be very heterogeneous. A study in a larger cohort would be necessary to draw clearer conclusions.

## 4. Discussion and Future Research

Our data point to an unusually high number of IIM cases progressively increasing during 2020, 2021, 2022, and 2023 in Spain, a highly vaccinated country, with specific MA patterns. Autoimmune and inflammatory pathologies have been associated with various infectious diseases, including COVID-19 [18]. In a previous study, our group identified a significant increase in anti-MDA5 and anti-ARS (PL-7) antibodies coinciding with the 2020 and 2021 COVID-19 pandemic years, respectively, based on LIA results [16]. Nevertheless, David P. et al. demonstrate an increase in new cases of anti-MDA5, yet other MSAs did not exhibit this striking pattern of increase. Interestingly, differences in antibody profile have been reported in different geographic groups during the pandemic [19]. For example, Asian populations have displayed higher frequencies of anti-ARS and anti-MDA5 antibodies than Caucasian populations [20].

Recent studies have indicated that the mortality rate of IIM accounts for 24% after a median follow-up period of 9.7 years, underscoring the importance of early diagnosis and treatment of these patients [21]. During the 2022 study period, the mortality rate in our cohort was 2.28% (n = 18). Of these deaths, 66.66% were associated with some type of oncological process, and the remaining cases in the context of autoimmune background and IIM. In recent years, an increased incidence of MAs and other new-onset autoimmune diseases has been reported, coinciding with the COVID-19 pandemic. We decided to conduct a retrospective, observational, and multicenter study from a routine diagnostic setting of the immunological characteristics and clinical spectra of IIM based on the array of MAs detected during the COVID-19 pandemic.

The identification of MAs, in conjunction with other complementary parameters, is of paramount importance for the diagnosis, prognosis, and treatment of IIM. The gold standard method for the detection of MAs is immunoprecipitation. However, there is no general consensus on the detection limits of MAs in the commercial methods used and there is controversy over their utility in IIM classification criteria. The detection of some antibodies, such as anti-MDA5, by immunoblotting correlates well with immunoprecipitation, but the detection of other antibodies, such as anti-OJ, by commercial methods has low sensitivity. This poses a challenge in routine practice due to the difficulty of interpreting the results. It is therefore crucial to emphasize the importance of laboratory work in antibody standardization. While the 2017 EULAR/ACR criteria consider only anti-Jo-1 as a biomarker for the diagnosis of IIM, in clinical practice it is important to evaluate other less frequent but significant MAs that can be detected through routine tests such as line dot immunoassays [22,23]. The exclusive reliance on anti-Jo-1 as a diagnostic criterion may result in the misdiagnosis of patients who would benefit from early intervention and improve outcomes with early immunosuppressive treatment.

In comparison to the prevalence of anti-PL-7 antibodies observed in the IIM population (<5%), our cohort exhibited an increased prevalence of 14.84%, which is higher than expected. A higher percentage of anti-PL-7 antibodies (38.37%) compared to anti-Jo-1 (26.74%) antibodies was also observed in Group I. This prompts the question of whether this change in the detected antibody profile, when compared to those described in the literature, could be related to a specific stimulus that may result in the release of cryptic epitopes expressed on antigen-presenting cells, which could potentially lead to an autoimmune response. Despite the described high prevalence of anti-Jo-1 antibodies in patients with PM (28–30%) [24], our cohort exhibited a very low prevalence of this condition, at 5.35%. Nevertheless, 33.33% of the cohort with anti-Jo-1 received a final diagnosis of ASSD.

The majority of MAs identified during 2022 belonged to Group II (MSA-non-ARS). The highest number of HPOS belonged to anti-Ro-52 MAA, with an overall prevalence of 24.42% in our cohort. MAAs can coexist with MSAs, with anti-Ro52 being one of the most common, helping to identify patients with more severe ILD and poorer outcomes [25].

Although there are studies indicating that LPOS should be interpreted with caution and many authors describe this as a possible clinical false positive result, it is important to interpret the results taking into account the patient’s clinical history. Our results demonstrated a higher frequency of anti-MDA5 and anti-SAE with LPOS associated with pulmonary involvement (17.16% and 16.57%, respectively). There are practically no long-term follow-up studies of these antibodies at LPOS. Most of the studies performed in patients with positive MAs are based on well-defined clinical phenotypes [26], as opposed to the presence of MAs in clinical groups with non-specific manifestations and long-term follow-up. However, a recent investigation indicates that patients with positive anti-MDA5 autoantibodies exhibit distinct phenotypic clusters. Those who survive the first three months, either by navigating the fulminant stage or beyond, may demonstrate progressive improvement in lung function and a favorable prognosis. In contrast, patients with anti-PL7 antibodies are more likely to experience a progressive loss of lung function and may have a higher subsequent mortality rate than other patient groups [27].

The prevalence of anti-TIF1-γ antibodies in DM has been reported to range from 7% to 41% [28], with an association with cancer in the range of 19 to 100%. In our cohort, the observed prevalence of anti-TIF1-γ was 27.90% associated with DM and 32.61% cancer-associated. It is noteworthy that 29.59% of patients with DM exhibited an associated oncologic process and 66.67% exhibited the anti-TIF1-γ antibody as the most frequently detected antibody, with a median age of 72 years (IQR: 77–57) and a male predominance. While anti-TIF1-γ and anti-NXP2 are the most common antibodies associated with neoplasia [29], Group II (MSA-non-ARS) has been the group with the highest frequency of this manifestation.

While the prevalence and clinical associations of specific autoantibodies like anti-TIF1-γ are important considerations, the choice of serological testing method can also impact the diagnosis of IIM. Some authors propose that ANA screening by HEp-2 IIF supports the diagnosis of IIM, while others argue that it is a technique with limitations due to insufficient sensitivity for some MSAs [30]. Our findings indicate that there is no significant difference in ANA positivity between the groups, which was 60.66%, in agreement with recent publications [20]. Statistically significant differences were observed for AC-19 and AC-4 immunofluorescence patterns between the groups (*p* < 0.05). In certain instances, the presence of characteristic HEp-2 and tissue patterns enhances the test’s specificity. This finding was also confirmed in the group of patients with SAID. Although no statistically significant differences were observed in antibody titers between final diagnosis groups (*p* < 0.05), the one with the highest percentage of strong positivity was the ASSD/ IIM-non-ASSD group. The ASSD/IIM-non-ASSD, SAID, and overlap IIM-SAID groups demonstrated greater consistency with positive ANA IIF, which supports the specificity of both techniques for clinical diagnosis with respect to the pre-ASSD/ IIM-non-ASSD and mixed/non-specific clinical features.

Recent studies have demonstrated that SARS-CoV-2 infection may increase the risk of developing another SAID in individuals with pre-existing autoimmune conditions [31]. It is notable that the incidence and prevalence of SAID vary across geographical regions. Overall, the prevalence of autoimmune diseases is estimated to be between 3 and 5% in the general population [32]. The prevalence of SAID in our cohort was 13.57%, which is more than double the rate observed in the general population. A recent review of 40 cases of COVID-19-associated myositis revealed that patients with a history of myositis concurrent with or developed post-infection comprised 78% of these cases, while myositis developed in the setting of vaccination comprised 22% of the cases. No cases of patients with a prior autoimmune disease history were included in the study [15]. Despite the lack of sufficient data to investigate potential associations between antibody positivity and the development of SAID in our group, 11.21% exhibited clinical onset post-pandemic. The remaining patients had already been diagnosed with SAID, thereby confirming the role of an underlying autoimmune background in the development of IIMs.

A recent publication has highlighted the coexistence of two or more MSAs among patients with IIM, with a prevalence of approximately 5% and a higher frequency of ILD. The presence of multiple MSAs complicates the classification of IIM and suggests a more complex autoimmune pathogenesis than previously thought [33]. Group III consisted of patients with multiple positivity, 24.85% of whom exhibited ILD. This group received the highest percentage of combined vaccines. The phenomenon of molecular mimicry, whereby vaccine antigens resemble self-antigens, has been postulated to play a role in the pathogenesis of SAID [12]. This is believed to occur through the activation of autoreactive T cells. It is postulated that adjuvants in vaccines share structural similarities with self-antigens, leading to cross-reactivity. Although the concepts of molecular mimicry and bystander activation are theoretical, the precise pathogenic processes linking vaccinations to autoimmune disorders remain unclear [34]. The distinct immune signatures elicited by the different vaccine combinations demonstrate that the immune response is shaped by the type of vaccines applied and the order in which they are delivered [35]. This could explain our observation in Group III. It is acknowledged that one of the limitations of this study is the absence of radiological and/or muscle biopsy data in many patients’ medical records. This precludes the performance of statistical analyses with these important diagnostic variables, which are part of current clinical practice for evaluating myositis. It should be noted, however, that clinical manifestations such as myositis may be the sole or initial presentation of IIM without involvement of other organs. This limitation underscores the lack of consensus across centers regarding cutoff points used to determine positivity for MAs. While the majority of centers employed manufacturers’ cutoff points as previously mentioned, there was no uniformity in practice. Specifically, some specialists only reported MA positivity if clinical signs of IIM were present, whereas others established their own cut-off points based on experience, which introduced inconsistencies when comparing positivity rates between patients seen at different centers. Consequently, the absence of diagnostic data and the inconsistency in MA cutoff interpretations present challenges for the study conclusions. Furthermore, due to the use of different commercial kits for antibody measurement, some of which simultaneously measured anti-Mi-2α/β and anti-PMScl-75/100 antibodies, there is a possibility that our results were overestimated. To address potential limitations associated with heterogeneous assay methodologies, future studies could aim to standardize assay protocols, thereby facilitating more robust comparisons across patient cohorts.

Our findings highlight the inherent complexity of autoimmune phenotypes, which presents both a challenge and an opportunity. While this complexity necessitates further stratification to improve diagnostic and prognostic outcomes, it also underscores the potential value of advanced approaches. In line with the innovative work of Hassler et al. (2024) [36], future research should consider applying clustering methodologies to identify distinct patient profiles within the spectrum of autoimmune myopathies. By incorporating such advanced analytical techniques, subsequent studies may not only validate our findings but also contribute to refining clinical classification criteria and therapeutic strategies.

This study presents our collective clinical experience and insights gained over the course of the COVID-19 pandemic. Despite ongoing efforts to standardize autoantibody testing, further progress is required to establish international cut-off values and reporting methods. Our preliminary findings suggest a potential correlation between SARS-CoV-2-induced myositis and MA positivity. This lends support to the hypothesis that viral immunological dysfunction, intrinsic predisposition, and unknown molecular triggers contribute to post-infection autoimmunity. However, one of the limitations of this work is not being able to compare these results with those obtained in the same time period before the pandemic. Further research is needed to determine the clinical relevance and magnitude of myositis–antibody associations in the post-COVID-19 context, while also elucidating potential mechanisms linking infection and/or vaccination, such as the homology between SARS-CoV-2 spike proteins and myositis antigens, particularly ARS.

Moreover, additional studies are needed to resolve key questions about the role of myositis autoantibodies (MAs) in the association between IIM and SARS-CoV-2:

1. How does the clinical pattern of IIM correlate with the specific type of antibody involved, especially in the presence of multiple antibodies?
2. Could the potential epitopes recognized by each antibody provide insight into the mechanisms underlying the association with COVID-19?
3. Does the persistence of these antibodies over time without associated clinical symptoms indicate a milder clinical phenotype or a true false positive?

## Figures and Tables

**Table 1:** Demographic, clinical, and laboratory characteristics of the study population. The chi-square test was used to compare categorical variables between groups. To indicate statistically significant differences between groups (*p* < 0.05), the symbol * was used, followed by the groups concerned. For example, *,1,2,3 indicates significant differences between Group I vs. II, Group I vs. III, and Group II vs. III. The results of some clinical and analytical parameters were calculated from the total data collected and not from the total number of patients.

	Total of Patients (n = 788)	Group I: MSA-ARS (n = 172)	Group II: MSA-Non-ARS (n = 443)	Group III: Multi-Positivity (n = 173)
**Demographic data**				
Females, n (%)	523 (66.37%)	105 (61%)	263 (59%)	115 (66%)
Age (years), mean (SD)	60.26 (14.80)	59.71 (12.65)	59.38 (11.34)	62.37 (12.06)
Mortality	18 (2.28%)	5 (2.90%)	12 (2.70%)	1 (0.57%)
**Laboratory data n (%)**				
Positivity of MAs	1209	221 (18.27%)	492 (40.69%)	496 (41.02%)
-HPOS	559 (46.23%)	143 (64.71%) *^,1,2,3^	259 (52.54%) *^,2,3^	157 (31.65%)
-LPOS	652 (53.76%)	78 (35.29%) *^,1,2,3^	234 (47.56%) *^,2,3^	338 (68.14%)
**ANA IIF positive (Hep-2)**	478 (60.66%)	109 (63.37%)	258 (58.247%)	111 (64.16%)
-AC-19/20 -AC-4		57 (52.29%) *^,1,2,3^ 49 (44.95%) *^,1,2,3^	31 (12.01%) 218 (84.49%)	23 (20.72%) 85 (73.87%)
**Presence of ENAs**	135 (17.13%)	34 (19.77%)	62 (13.99%) *^,2,3^	39 (22.54%)
**Enzymes value median (interquartile range)**				
CK (10–155 U/L)	91 (56–159)	92.5 (57–169)	91 (57–162)	90 (56–156)
Aldolase (<17.5 U/L)	6 (4–8)	5.5 (4–9)	5.45 (4–7)	5.8 (4–8)
ALT (0–35 U/L)	23 (18–31)	20 (14–30)	19.5 (14–30)	20 (15–30)
AST (0–35 U/L)	20 (14–30)	22 (19–30)	22 (18–31)	24 (19–32)
LDH (208–308 U/L)	259 (184–347)	217 (186–309)	218 (180–328)	318 (222–414)
**Clinical Features n (%)**				
-Asthenia	260 (32.99%)	68 (39.53%) *^,1,2^	125 (28.21%) *^,2,3^	67 (38.72%)
-Muscle	269 (34.57%)	67 (38.95%)	141 (31.82%)	61 (35.26%)
-Lung	313 (39.72%)	81 (47.09%) *^,1,2^	155 (34.98%) *^,2,3^	77 (44.50%)
-Skin	150 (19.03%)	35 (20.34%)	90 (20.31%)	25 (14.45%)
-Neoplasia	102 (12.94%)	19 (11.04%) *^1,2,3^	80 (18.05%) *^,2,3^	3 (1.73%)
**Final diagnosis n (%)**				
-ASSD/IIM-non-ASSD	168 (21.31%)	54 (31.39%) *^,1,2^	73 (16.47%) *^,2,3^	41 (26.69%)
-Pre-ASS/Pre-IIM-non-ASSD	54 (6.85%)	20 (11.62%) *^,1,2^	21 (4.74%)	13 (7.51%)
-SAID	107 (13.57%)	15 (8.72%) *^,1,3^	62 (13.99%)	30 (17.34%)
-Overlap IIM/SAID	38 (4.82%)	15 (8.72%) *^,1,2,3^	17 (3.83%)	6 (3.46%)
-Mixed/non specific clinical features	421 (53.42%)	* 68 (39.53%) *^,1,2^	270 (60.94%) *^,2,3^	83 (47.97%)
**Treatment n (%)**	240 (30.45%)	57 (33.13%)	126 (28.44%)	57 (32.94%)
-Prednisone	145 (18.40%)	36 (20.93%)	74 (16.70%)	35 (20.23%)
-Hydroxychloroquine	36 (4.56%)	6 (3.48%)	18 (4.06%)	12 (6.93%)
-MTX	33 (4.18%)	7 (4.06%)	19 (4.28%)	7 (4.04%)
-Antifibrotic	15 (1.90%)	2 (1.16%)	6 (1.35%)	7 (4.04%)
-Biologic	57 (7.23%)	22 (12.79%)	27 (6.09%)	8 (4.62%)
-IVIg	18 (2.28%)	2 (1.16%)	12 (2.70%)	6 (3.46%)
**Relationship between symptoms or autoantibody positivity and COVID19 infection/vaccination n (%)**				
Infection NO Vaccine NO	39 (4.98%)	2 (1.16%)	33 (7.45%)	4 (2.31%)
Infection YES Vaccine NO	24 (3.07%)	3 (1.74%)	14 (3.16%)	7 (4.05%)
Infection NO Vaccine YES	445 (56.83%)	108 (62.79%) *^,1,3^	249 (56.21%)	88 (50.87%)
Infection YES Vaccine YES	275 (35.12%)	57 (33.14%)	144 (32.51%)	74 (42.77%)
Infection preceding symptoms	141 (47.16%)	24 (13.95%) *^,1,3^	71 (16.03%)	* 46 (26.59%)
* Vaccination preceding symptoms	312 (43.33%)	74 (43.02%)	162 (36.57%)	76 (43.93%)
mARN Vaccine	517 (71.81%)	122 (73.93%)	267 (67.93%) *^,2,3^	128 (79.01%)
no-mARN Vaccine	95 (13.19%)	18 (12.41%) *^,1,3^	71 (19.51%)	6 (3.75%)
Combinated vaccination	57 (79.17%)	5 (3.44%) *^,1,3^	26 (7.14%) *^,2,3^	26 (16.25%)
Vaccine number of doses (median ± SD)	3.0 ± 1.16	3.0 ± 1.14	3.0 ± 1.86	3.0 ± 1.14

**Table 2:** Profile of myositis autoantibodies in relation to final diagnosis and COVID-19 status. The number (n) and percentage (%) of patients in each category were used to describe categorical variables. The first column “frequency of MAs” lists the three most frequent antibodies in each group. Under “Infection Preceding Symptoms”, the total number of infected patients was considered, whereas under “Vaccination Preceding Symptoms,” only vaccinated patients without documented infection were included.

	ASSD/IIM-Non-ASSD (n = 168)	Pre- ASSD/Pre-IIM-Non-ASSD (n = 54)	SAID (n = 107)	Overlap IIM-SAID (n = 38)	Non-Specific (n = 421)
Frequency of MAs (n)	anti-Ro52 (n = 48) anti-Mi2α/β (n = 30) anti-PL7 (n = 29)	anti-Jo1 (n = 10) anti-ku (n = 9) anti-PL-7 (n = 8)	anti-Ro52 (n = 40) anti-PMScl-75/100 (n = 28) anti-Mi2α/β (n = 23)	anti-Ro52 (n = 13) anti-PMScl (n = 9) anti-PL7 (n = 7)	anti-Mi2α/β (n = 81) anti- PMScl-75/100 (n = 81) anti-Ro52 (n = 72)
HPOS n (%)	63 (58.33%)	13 (24.07%)	37 (34.57%)	16 (42.10%)	142 (33.72%)
ANA n (%)	117 (69.64%)	32 (59.25%)	81 (75.70%)	28 (73.68%)	220 (52.25%)
Infection preceding symptoms n (%)	25 (14.88%)	16 (29.62%)	44 (41.12%)	6 (15.78%)	75 (17.81%)
Vaccination preceding symptoms n (%)	50 (47.16%)	10 (43.47%)	44 (44.89%)	9 (47.36%)	100 (41.84%)

## Data Availability

The original contributions presented in this study are included in the article. Further inquiries can be directed to the corresponding author.

## Abbreviations

ANA	antinuclear antibodies
ARS	aminoacyl-tRNAsynthetase
ASSD	antisynthetase syndrome
COVID-19	coronavirus disease 2019
CK	creatine kinase
DM	dermatomyositis
ENA	extractable nuclear antigen
HEp-2	human epithelial cell line
HLA	human leucocyte antigen
HPOS	high positive
IIF	indirect immunofluorescence
ILD	interstitial lung disease
IIM	idiopathic inflammatory myopathies
LIA	line blot immunoassays
LPOS	low positive
MA	myositis autoantibodies
MAA	myositis-associated autoantibodies
MCP	mycophenolate mofetil
Mrna	messenger RNA
MSA	myositis-specific autoantibodies
PM	polymyositis
RA	rheumatoid arthritis
RT-PCR	reverse transcription polymerase chain reaction
SAID	systemic autoimmune diseases
SARS-CoV-2	severe-acute-respiratory-syndrome-related coronavirus
SLE	systemic lupus erythematosus
SjS	Sjögren syndrome
SSc	systemic sclerosis
